# Generative artificial intelligence literacy profiles differ in academic self-efficacy and subjective well-being among adolescents

**DOI:** 10.3389/fpsyg.2026.1987633

**Published:** 2026-10-14

**Authors:** Ja-Yeoung Lee

**Affiliations:** Department of Nursing, Dongguk University WISE Campus, Gyeongju, Republic of Korea

**Keywords:** academic self-efficacy, adolescents, artificial intelligence, cluster analysis, generative AI literacy, subjective well-being

## Abstract

Generative artificial intelligence (AI) literacy is increasingly important for adolescents, yet variation in literacy across multiple dimensions and its associations with educational and psychological outcomes remain insufficiently understood. This study identified generative AI literacy profiles among Korean adolescents with generative AI experience (*N* = 3,959) using K-means cluster analysis based on information evaluation, communication, creative use, and ethical use. Two profiles were identified: Higher AI Literacy and Lower AI Literacy. The profiles differed primarily in their overall literacy levels rather than in qualitatively distinct patterns across the four dimensions. Adolescents in the Higher AI Literacy profile reported greater generative AI education experience and higher academic self-efficacy and subjective well-being than those in the Lower AI Literacy profile. Differences in academic self-efficacy and subjective well-being remained significant after adjustment for gender, school level, academic achievement, and economic status. These findings demonstrate meaningful level-based variation in generative AI literacy among adolescents with generative AI experience and suggest that such variation is associated with educational experience and academic and psychological outcomes. A person-centred perspective may complement variable-centred approaches and inform future research and educational approaches that consider differences in students’ generative AI literacy levels.

## 1 Introduction

The rapid diffusion of generative artificial intelligence (AI) is transforming adolescents’ learning environments and information-use practices. Generative AI tools such as ChatGPT are increasingly used for information seeking, assignment completion, writing, and problem solving, leading to greater engagement by adolescents in their everyday learning experiences (Kasneci et al., 2023; UNESCO, 2023). While these developments provide new learning opportunities, they also underscore the importance of competencies required to critically evaluate and responsibly use AI-generated information.

In this context, generative AI literacy has emerged as a key competency for navigating AI-mediated learning environments. Generative AI literacy encompasses not only the ability to understand and use generative AI tools but also the ability to evaluate the accuracy and credibility of AI-generated information and utilise such technologies ethically and responsibly (Long and Magerko, 2020; Ng et al., 2021a, 2021b). Recent studies suggest that generative AI literacy is positively associated with academic performance and learning experiences (Bećirović et al., 2025), indicating that students with higher levels of AI-related competence may be better equipped to engage with AI-supported learning and academic tasks. Generative AI literacy may therefore be understood not only as a technological competency but also as an educational resource associated with students’ learning and adaptation. In the present study, generative AI literacy is conceptualised as a multidimensional construct encompassing information evaluation, communication, creative use, and ethical use, reflecting competencies relevant to adolescents’ engagement with generative AI in educational contexts.

The significance of generative AI literacy may also extend beyond learning-related outcomes. As generative AI becomes increasingly integrated into educational settings, differences in students’ ability to use and critically evaluate AI may be associated with their academic and psychological adjustment. Academic self-efficacy, which reflects students’ confidence in their ability to successfully perform academic tasks, may be particularly relevant in this context (Bandura, 1997; Pajares, 1996). Students who are better able to use generative AI for information seeking and problem solving may experience greater confidence when engaging with academic tasks. Such differences may also be associated with subjective well-being, an important indicator of adolescents’ psychological adjustment (Castelli and Marcionetti, 2024; Cherewick et al., 2024). Thus, examining academic self-efficacy and subjective well-being across levels of generative AI literacy may provide insight into the broader educational and psychological significance of adolescents’ AI-related competencies.

Despite its growing importance, much of the existing research on generative AI literacy has focused on average-level relationships with educational and psychological outcomes (Bećirović et al., 2025). Such variable-centred analyses may not adequately capture heterogeneity in adolescents’ experiences with and competencies related to generative AI. Person-centred approaches can identify distinct subgroups characterised by different patterns of competencies and experiences, offering a deeper understanding of individual differences (Bergman and Trost, 2006; Howard and Hoffman, 2018). Recent studies have begun to demonstrate such heterogeneity. Studies of digital literacy have identified distinct student profiles associated with differences in learning-related outcomes (Jeong and Choi, 2024; Kil et al., 2023), while Wang et al. (2026) identified distinct AI literacy profiles among K–12 students. Together, these findings suggest that AI-related competencies are heterogeneous and that distinct literacy profiles may emerge across different student populations and educational contexts.

However, important gaps remain in the existing profile literature. Previous Korean studies have primarily examined profiles of general digital literacy and their associations with learning-related outcomes or predictors of profile membership (Jeong and Choi, 2024; Kil et al., 2023). More recently, Wang et al. (2026) identified profiles of broader AI literacy among Chinese K–12 students and examined their predictors and differences in self-regulated learning. These studies establish the value of person-centred approaches but do not specifically address literacy in the context of generative AI use. Generative AI presents distinctive demands related to evaluating AI-generated information, communicating effectively with generative AI, using it creatively, and addressing ethical issues arising from its use. Moreover, little is known about whether profiles across these dimensions are associated with adolescents’ academic self-efficacy and subjective well-being. The present study extends prior profile research by focusing specifically on adolescents with generative AI experience and examining profiles across information evaluation, communication, creative use, and ethical use, together with differences in educational experience and indicators of academic and psychological adjustment. Importantly, the person-centred approach was used to examine whether meaningful configurations emerged across these four dimensions without presupposing that profiles would necessarily differ qualitatively in shape rather than primarily in overall level.

Accordingly, this study aimed to identify generative AI literacy profiles among adolescents with experience using generative AI and to examine differences in background characteristics, generative AI education experience, academic self-efficacy, and subjective well-being across the identified profiles. By adopting a person-centred approach, this study extends existing research beyond variable-centred analyses of average-level associations by examining whether adolescents can be differentiated according to their patterns of generative AI literacy across multiple dimensions. This approach may provide additional insight into variation in adolescents’ generative AI literacy and its associations with educational and psychological outcomes. The findings may inform differentiated educational support responsive to students’ varying levels of generative AI literacy.

Research Questions.

*RQ1*. What generative AI literacy profiles can be identified among adolescents based on the four dimensions of information evaluation, communication, creative use, and ethical use?

*RQ2*. Do adolescents’ background characteristics differ across the identified generative AI literacy profiles?

*RQ3*. Do generative AI education experience, academic self-efficacy, and subjective well-being differ across the identified generative AI literacy profiles?

## 2 Materials and methods

### 2.1 Study design and participants

This study was a cross-sectional secondary data analysis aimed at identifying generative AI literacy profiles among adolescents and examining differences in generative AI education experience, academic self-efficacy, and subjective well-being across these profiles. Data were obtained from the 2024 Study on Adolescents’ Use of Generative AI and Strategies for Enhancing AI Literacy conducted by the National Youth Policy Institute (NYPI) (Lee et al., 2024).

The original survey was conducted from May to July 2024 using a stratified multistage cluster sampling design based on region, school level, school type, and grade. One class was randomly selected from each sampled school, and all students in the selected class completed a self-administered questionnaire during school hours. A total of 5,778 middle and high school students in South Korea participated in the survey. Among them, 1,819 adolescents who reported no experience using generative AI were excluded because the present study focused on literacy profiles among adolescents with prior generative AI experience. The final analytical sample therefore comprised 3,959 adolescents.

### 2.2 Measures

#### 2.2.1 Generative AI literacy

Generative AI literacy was measured using a 12-item scale adapted from the ChatGPT Literacy Scale developed by Lee and Park (2023). The measure comprised four subdomains: information evaluation, communication, creative use, and ethical use. Information evaluation assessed the ability to evaluate the credibility and accuracy of AI-generated information. Communication assessed the ability to communicate effectively using generative AI. Creative use assessed the ability to generate new ideas and creative outputs using generative AI. Ethical use assessed awareness of privacy protection, copyright, and the ethical and responsible use of generative AI.

Each subdomain consisted of three items, and all items were rated on a 5-point Likert scale ranging from 1 (strongly disagree) to 5 (strongly agree). Mean scores were calculated for each subdomain, with higher scores indicating greater generative AI literacy. In the present study, the internal consistency reliability of the overall 12-item scale was excellent (Cronbach’s α = 0.927).

#### 2.2.2 Generative AI education experience

Generative AI education experience was measured using a four-item instrument developed for the original survey (Lee et al., 2024). The instrument assessed educational experiences related to understanding how generative AI works, learning how to use generative AI, receiving education on privacy and copyright issues associated with generative AI, and identifying errors and biases in AI-generated information. Each item was rated on a 4-point Likert scale ranging from 1 (never received) to 4 (frequently received). Mean item scores were calculated, with higher scores indicating greater generative AI education experience. In the present study, the internal consistency reliability of the scale was excellent (Cronbach’s α = 0.936).

#### 2.2.3 Academic self-efficacy

Academic self-efficacy was measured using a six-item scale adapted from Bulfone et al. (2020). The scale assessed confidence in academic performance and problem-solving ability. Each item was rated on a 5-point Likert scale ranging from 1 (strongly disagree) to 5 (strongly agree). Mean item scores were calculated, with higher scores indicating greater academic self-efficacy. In the present study, the internal consistency reliability of the scale was excellent (Cronbach’s α = 0.907).

#### 2.2.4 Subjective well-being

Subjective well-being was measured using a six-item scale adapted from Alshallal et al. (2024). The scale assessed hope for the future, peer relationships, happiness, expectations for daily life, meaning in life, and enjoyment of everyday life. Each item was rated on a 5-point Likert scale ranging from 1 (strongly disagree) to 5 (strongly agree). Mean item scores were calculated, with higher scores indicating greater subjective well-being. In the present study, the internal consistency reliability of the scale was excellent (Cronbach’s α = 0.929).

### 2.3 Data analysis

The data were analysed using IBM SPSS Statistics version 29.0 and IBM SPSS Amos version 32. Descriptive statistics, including frequencies, percentages, means, and standard deviations, were calculated to describe participant characteristics and study variables.

Confirmatory factor analysis (CFA) was conducted to evaluate the construct validity of the 12-item generative AI literacy measure using a four-factor model comprising information evaluation, communication, creative use, and ethical use. Model fit was evaluated using the chi-square statistic (χ^2^), comparative fit index (CFI), Tucker–Lewis index (TLI), and root mean square error of approximation (RMSEA). Convergent validity was evaluated using standardized factor loadings, composite reliability (CR), and average variance extracted (AVE). Discriminant validity was assessed by comparing the square root of the AVE for each factor with the corresponding inter-factor correlations.

K-means cluster analysis was conducted using the four generative AI literacy subdomain scores to identify profiles of adolescents with similar literacy patterns. There were no missing data for the variables included in the cluster analysis; therefore, no missing-data imputation or additional case exclusion was required. Cluster analysis was used as a person-centred approach to examine heterogeneity in generative AI literacy (Bergman and Trost, 2006; MacQueen, 1967). Solutions ranging from two to five clusters were evaluated using the within-cluster sum of squares (WSS), silhouette coefficient, Calinski–Harabasz index, and cluster stability. Cluster stability was assessed using 200 iterations of 80% subsampling and quantified using the adjusted Rand index (ARI). The final number of clusters was determined by considering cluster separation, stability, and parsimony.

Differences in gender, school level, academic achievement, and economic status between the identified profiles were examined using chi-square (*χ*^2^) tests, with Cramér’s V reported as an effect-size measure. Differences in generative AI education experience between profiles were examined using Welch’s analysis of variance because the homogeneity-of-variance assumption was violated. Hedges’ g and its 95% confidence interval were calculated to quantify the magnitude of the between-profile difference.

Differences in academic self-efficacy and subjective well-being between profiles were examined using general linear models adjusting for gender, school level, academic achievement, and economic status. Adjusted means with 95% confidence intervals and partial η^2^ were reported. Because Levene’s tests indicated heterogeneity of variance for both outcomes, sensitivity analyses using heteroskedasticity-consistent HC3 standard errors were additionally conducted to assess the robustness of the profile differences.

The original survey employed a complex sampling design and provided sampling weights. Because K-means clustering is a distance-based, unsupervised classification procedure and does not directly incorporate survey weights in the standard SPSS implementation, the primary profile identification was conducted using unweighted data. As a sensitivity analysis, the standardized sampling weight provided with the dataset was subsequently applied to examine the weighted distribution of the identified profiles, their four generative AI literacy dimensions, and between-profile differences in the study outcomes. The weighted analyses were compared with the primary unweighted results to assess the robustness of the findings. Statistical significance was set at *p* < 0.05.

## 3 Results

### 3.1 Participant characteristics

A total of 3,959 adolescents with experience using generative AI were included in the final analysis (Table 1). Of the participants, 2,191 (55.3%) were male, and 1,768 (44.7%) were female. Regarding school level, 2,181 participants (55.1%) were high school students and 1,778 (44.9%) were middle school students.

**Table 1:** Characteristics of participants (*N* = 3,959).

Characteristics	n	%
Gender		
Male	2,191	55.3
Female	1,768	44.7
School level		
Middle school	1,778	44.9
High school	2,181	55.1
Academic achievement		
Very low	174	4.4
Low	576	14.5
Average	1,724	43.5
High	973	24.6
Very high	512	12.9
Economic status		
Low	96	2.4
Average	3,024	76.4
High	839	21.2

For academic performance, the largest proportion of participants reported an average level (*n* = 1,724, 43.5%), followed by high (*n* = 973, 24.6%), low (*n* = 576, 14.5%), very high (*n* = 512, 12.9%), and very low (*n* = 174, 4.4%) levels. Regarding perceived economic status, most participants reported an average level (*n* = 3,024, 76.4%), followed by a high level (*n* = 839, 21.2%) and a low level (*n* = 96, 2.4%). Detailed participant characteristics are presented in Table 1.

### 3.2 Levels of the study variables

The levels of generative AI education experience, generative AI literacy, academic self-efficacy, and subjective well-being are presented in Table 2. The mean scores for generative AI education experience, generative AI literacy, academic self-efficacy, and subjective well-being were 2.337 (SD = 0.847), 3.484 (SD = 0.802), 3.389 (SD = 0.930), and 3.774 (SD = 0.916), respectively.

**Table 2:** Means and standard deviations of study variables.

Variables	Mean	SD
AI education experience	2.337	0.847
Generative AI literacy	3.484	0.802
Information evaluation	3.473	0.975
Communication	3.537	0.965
Creative use	3.424	0.961
Ethical use	3.503	0.965
Academic self-efficacy	3.389	0.930
Subjective well-being	3.774	0.916

Among the four subdomains of generative AI literacy, communication had the highest mean score (*M* = 3.537, SD = 0.965), followed by ethical use (*M* = 3.503, SD = 0.965), information evaluation (*M* = 3.473, SD = 0.975), and creative use (*M* = 3.424, SD = 0.961).

### 3.3 Construct validity of the generative AI literacy measure

Confirmatory factor analysis was conducted to evaluate the construct validity of the 12-item generative AI literacy measure, comprising four correlated factors: information evaluation, communication, creative use, and ethical use. The four-factor model demonstrated good fit to the data, *χ*^2^(48) = 463.06, *p* < 0.001, CFI = 0.987, TLI = 0.983, and RMSEA = 0.047, 90% CI [0.043, 0.051]. Standardized factor loadings ranged from 0.787 to 0.911, and all loadings were statistically significant (*p* < 0.001). Composite reliability values ranged from 0.866 to 0.900, while average variance extracted (AVE) values ranged from 0.684 to 0.751, supporting convergent validity. In addition, the square roots of the AVEs (0.827–0.867) exceeded the corresponding inter-factor correlations (0.513–0.736), providing evidence of discriminant validity among the four dimensions. Detailed factor loadings, reliability and validity indices, and inter-factor correlations are presented in Table 3.

**Table 3:** Confirmatory factor analysis and construct validity of the generative AI literacy measure.

Factor	Item	Standardized loading	CR	AVE
Panel A. standardized factor loadings, composite reliability, and average variance extracted				
Information evaluation	Q14_1_1	0.855	0.900	0.751
	Q14_1_2	0.895		
	Q14_1_3	0.849		
Communication	Q14_2_4	0.854	0.888	0.726
	Q14_2_5	0.787		
	Q14_2_6	0.911		
Creative use	Q14_3_7	0.854	0.870	0.691
	Q14_3_8	0.818		
	Q14_3_9	0.821		
Ethical use	Q14_4_10	0.840	0.866	0.684
	Q14_4_11	0.815		
	Q14_4_12	0.825		

Factor	1	2	3	4
Panel B. inter-factor correlations and discriminant validity				
1. Information evaluation	**0.867**			
2. Communication	0.670	**0.852**		
3. Creative use	0.513	0.707	**0.831**	
4. Ethical use	0.650	0.736	0.688	**0.827**

*N* = 3,959. CR = composite reliability; AVE = average variance extracted. Diagonal values in Panel B are the square roots of AVE (√AVE); off-diagonal values are inter-factor correlations.

Model fit: *χ*^2^(48) = 463.06, *p* < 0.001, CFI = 0.987, TLI = 0.983, RMSEA = 0.047, 90% CI [0.043, 0.051]. Bold diagonal values indicate the square roots of AVE (√AVE).

### 3.4 Identification of generative AI literacy profiles

K-means cluster analyses were conducted using the four dimensions of generative AI literacy: information evaluation, communication, creative use, and ethical use. Solutions ranging from two to five clusters were evaluated using the within-cluster sum of squares (WSS), silhouette coefficient, Calinski–Harabasz index, and cluster stability (Table 4). WSS decreased across solutions, from 8,405.83 for two clusters to 5,049.99 for five clusters. The two-cluster solution yielded the highest silhouette coefficient (0.362), Calinski–Harabasz index (3,006.82), and cluster stability (mean ARI = 0.998). Considering cluster separation, stability, and parsimony, the two-cluster solution was retained.

**Table 4:** Comparison of alternative K-means cluster solutions.

Number of clusters	WSS	Silhouette coefficient	Calinski–Harabasz index	Stability (mean ARI)
2	8,405.83	**0.362**	**3,006.82**	**0.998**
3	6,643.97	0.319	2,424.72	**0.**987
4	5,944.74	0.261	1,962.52	0.975
5	5,049.99	0.269	1,906.87	0.955

WSS = within-cluster sum of squares; ARI = adjusted Rand index. Stability was assessed using 200 iterations of 80% subsampling. Bold values indicate the retained two-cluster solution.

The Higher AI Literacy profile comprised 1,867 adolescents (47.2%) and showed consistently higher scores for information evaluation (*M* = 4.15, SD = 0.70), communication (*M* = 4.25, SD = 0.60), creative use (*M* = 4.03, SD = 0.74), and ethical use (*M* = 4.19, SD = 0.66). The Lower AI Literacy profile comprised 2,092 adolescents (52.8%) and showed lower scores for information evaluation (*M* = 2.87, SD = 0.77), communication (*M* = 2.90, SD = 0.75), creative use (*M* = 2.89, SD = 0.81), and ethical use (*M* = 2.89, SD = 0.76). As illustrated in Figure 1, the profiles primarily differed in their overall levels of generative AI literacy rather than exhibiting qualitatively distinct patterns across the four dimensions.

**Figure 1:**
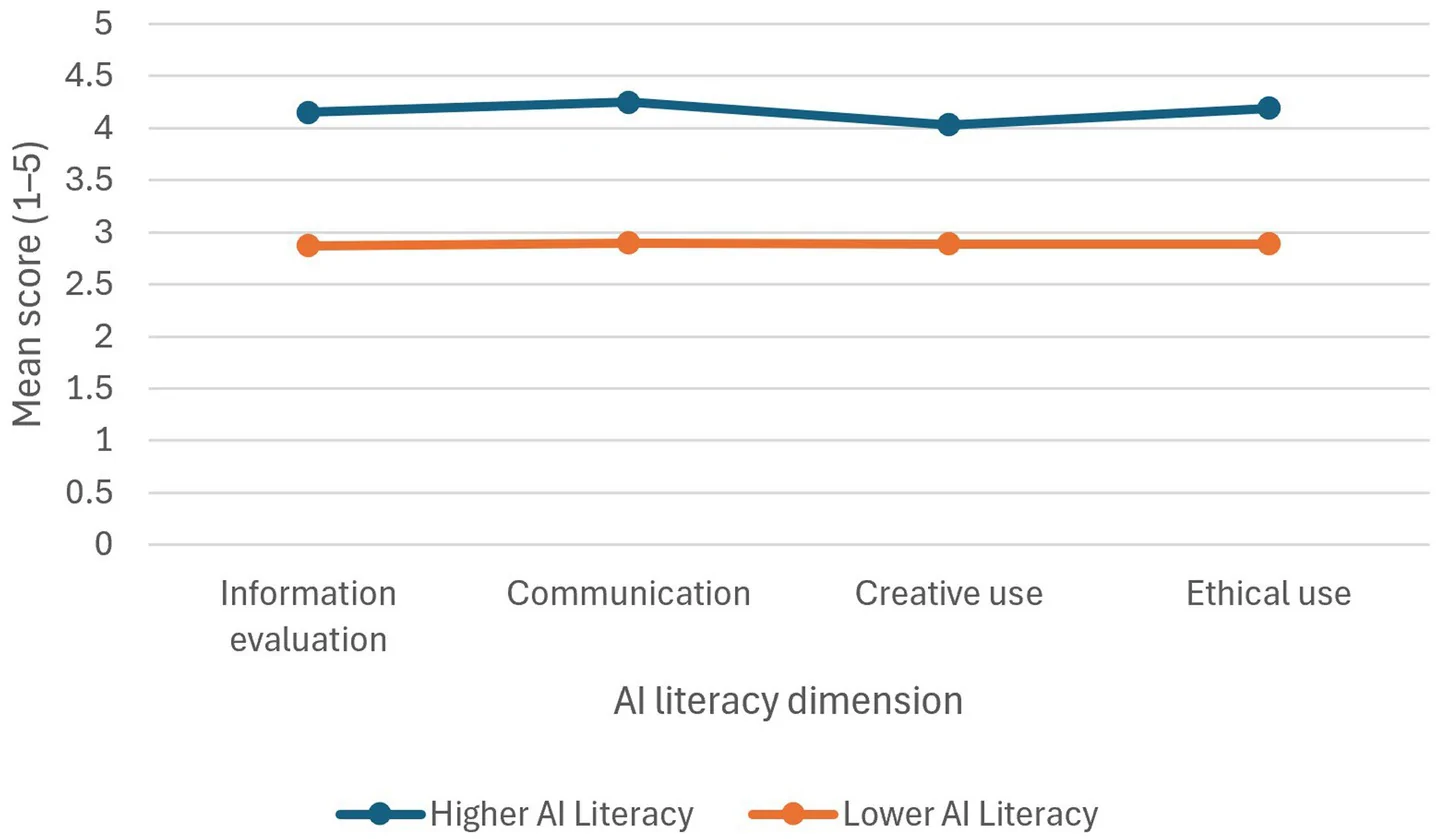
Generative AI literacy profiles across the four dimensions. Values represent mean scores for each generative AI literacy dimension. Higher AI Literacy, *n* = 1,867; Lower AI Literacy, *n* = 2,092.

### 3.5 Differences in background characteristics across profiles

Significant differences in background characteristics were observed between the two generative AI literacy profiles (Table 5). Gender distribution differed significantly by profile, *χ*^2^(1) = 57.85, *p* < 0.001, Cramér’s V = 0.121, with males comprising 61.7% of the Higher AI Literacy profile and 49.7% of the Lower AI Literacy profile. School level also differed significantly, *χ*^2^(1) = 27.87, *p* < 0.001, Cramér’s V = 0.084; high school students comprised 59.5% of the Higher AI Literacy profile compared with 51.1% of the Lower AI Literacy profile.

**Table 5:** Background characteristics by generative AI literacy profile.

Characteristic	Higher AI literacy *n* (%)	Lower AI literacy *n* (%)	*χ* ^2^	*p*	Cramér’s *V*
Gender			57.85	< 0.001	0.121
Male	1,152 (61.7)	1,039 (49.7)			
Female	715 (38.3)	1,053 (50.3)			
School level			27.87	< 0.001	0.084
Middle school	756 (40.5)	1,022 (48.9)			
High school	1,111 (59.5)	1,070 (51.1)			
Academic achievement			142.09	< 0.001	0.189
Very low	46 (2.5)	128 (6.1)			
Low	201 (10.8)	375 (17.9)			
Average	761 (40.8)	963 (46.0)			
High	547 (29.3)	426 (20.4)			
Very high	312 (16.7)	200 (9.6)			
Economic status			18.01	< 0.001	0.067
Low	38 (2.0)	58 (2.8)			
Average	1,381 (74.0)	1,643 (78.5)			
High	448 (24.0)	391 (18.7)			

Higher AI Literacy, *n* = 1,867; Lower AI Literacy, *n* = 2,092. Percentages are calculated within each profile.

Academic achievement differed significantly between profiles, *χ*^2^(4) = 142.09, *p* < 0.001, Cramér’s V = 0.189. The Higher AI Literacy profile had greater proportions of students reporting high (29.3%) or very high (16.7%) academic achievement than the Lower AI Literacy profile (20.4 and 9.6%, respectively). Economic status also differed significantly, *χ*^2^(2) = 18.01, *p* < 0.001, Cramér’s V = 0.067; 24.0% of the Higher AI Literacy profile reported high economic status compared with 18.7% of the Lower AI Literacy profile.

### 3.6 Differences in generative AI education experience across profiles

Generative AI education experience differed significantly between the two literacy profiles. Adolescents in the Higher AI Literacy profile reported greater generative AI education experience (*M* = 2.55, SD = 0.91) than those in the Lower AI Literacy profile (*M* = 2.15, SD = 0.74). Because the assumption of homogeneity of variance was violated, Levene’s *F*(1, 3,957) = 102.17, *p* < 0.001, Welch’s test was used. The difference between profiles was statistically significant, Welch’s *F*(1, 3599.41) = 221.25, *p* < 0.001, with Hedges’ g = 0.48, 95% CI [0.42, 0.54].

### 3.7 Differences in academic self-efficacy and subjective well-being across profiles

Differences in academic self-efficacy and subjective well-being between the two generative AI literacy profiles were examined after adjusting for gender, school level, academic achievement, and economic status. For academic self-efficacy, the adjusted mean was significantly higher in the Higher AI Literacy profile [Madj = 3.65, 95% CI (3.58, 3.72)] than in the Lower AI Literacy profile [Madj = 2.96, 95% CI (2.90, 3.03)], *F*(1, 3,949) = 696.83, *p* < 0.001, partial η^2^ = 0.150. The adjusted mean difference was 0.69 [95% CI (0.64, 0.74)].

A similar pattern was observed for subjective well-being. The Higher AI Literacy profile had a significantly higher adjusted mean [Madj = 3.82, 95% CI (3.75, 3.89)] than the Lower AI Literacy profile [Madj = 3.37, 95% CI (3.31, 3.44)], *F*(1, 3,949) = 257.24, *p* < 0.001, partial η^2^ = 0.061. The adjusted mean difference was 0.45 [95% CI (0.39, 0.50)].

Because Levene’s tests were significant for both academic self-efficacy and subjective well-being (*p* < 0.001), heteroskedasticity-consistent HC3 standard errors were additionally used in sensitivity analyses. The profile differences remained statistically significant for academic self-efficacy [B = 0.69, SE = 0.03, 95% CI (0.64, 0.74), *p* < 0.001] and subjective well-being [B = 0.45, SE = 0.03, 95% CI (0.39, 0.50), *p* < 0.001], supporting the robustness of the findings. Results for generative AI education experience and the adjusted comparisons of academic self-efficacy and subjective well-being are summarized in Table 6.

**Table 6:** Differences in study outcomes by generative AI literacy profile.

Outcome	Higher AI literacy	Lower AI literacy	Statistic	*p*	Effect size
Generative AI education experienceᵃ	2.55 (0.91)	2.15 (0.74)	Welch’s *F* = 221.25	< 0.001	*g* = 0.48 [0.42, 0.54]
Academic self-efficacyᵇ	3.65 [3.58, 3.72]	2.96 [2.90, 3.03]	*F* = 696.83	< 0.001	η_p_^2^ = 0.150
Subjective well-beingᵇ	3.82 [3.75, 3.89]	3.37 [3.31, 3.44]	*F* = 257.24	< 0.001	η_p_^2^ = 0.061

AI education values are M (SD); other values are adjusted means [95% CI]. ᵃ Hedges’ g [95% CI]. ᵇ Adjusted for gender, school level, academic achievement, and economic status.

Sensitivity analyses using the standardized sampling weight yielded substantively similar results. The weighted proportions of the Higher and Lower AI Literacy profiles were 46.0 and 54.0%, respectively, compared with 47.2 and 52.8% in the unweighted analysis, and the pattern of the four literacy dimensions remained essentially unchanged. Between-profile differences also remained significant for generative AI education experience, Welch’s *F*(1, 3477.09) = 209.81, *p* < 0.001. In the covariate-adjusted weighted general linear models, the profile effect remained significant for academic self-efficacy, *F*(1, 3,844) = 652.43, *p* < 0.001, partial η^2^ = 0.145, and subjective well-being, *F*(1, 3,844) = 204.04, *p* < 0.001, partial η^2^ = 0.050.

## 4 Discussion

This study identified two generative AI literacy profiles among adolescents with experience using generative AI: Higher AI Literacy and Lower AI Literacy. The two profiles differed consistently across information evaluation, communication, creative use, and ethical use, with the differences primarily reflecting overall levels of generative AI literacy rather than qualitatively distinct patterns across the four dimensions. Significant differences in profile membership were also observed according to gender, school level, academic achievement, and economic status. Adolescents in the Higher AI Literacy profile reported greater generative AI education experience than those in the Lower AI Literacy profile. Moreover, after adjusting for these background characteristics, the Higher AI Literacy profile showed higher academic self-efficacy and subjective well-being than the Lower AI Literacy profile, and these differences remained robust in sensitivity analyses. Analyses using the standardized sampling weight also yielded substantively similar findings. Overall, these results indicate meaningful variation in generative AI literacy levels among adolescents with generative AI experience and show that the identified profiles are associated with differences in educational experience, academic self-efficacy, and subjective well-being.

Wang et al. (2026) identified four latent AI literacy profiles among Chinese K–12 students and reported differences in self-regulated learning across profiles, providing evidence that students may vary in their configurations of AI-related competencies. The present study extends this line of research to generative AI literacy and identified two profiles, Higher AI Literacy and Lower AI Literacy. Unlike profiles characterised by qualitatively different configurations across dimensions, however, the two profiles identified in this study showed largely parallel patterns across information evaluation, communication, creative use, and ethical use and were distinguished primarily by their overall levels of generative AI literacy. Differences between the profile structures identified by Wang et al. (2026) and the present study may reflect differences in the populations, constructs and measurement instruments, and analytical approaches employed; Wang et al. used latent profile analysis to examine broader AI literacy, whereas the present study used K-means clustering to examine four dimensions specifically related to generative AI literacy. Thus, the present findings support variation in adolescents’ generative AI literacy while also indicating that, in this sample, such variation was expressed predominantly as differences in overall literacy level rather than as qualitatively distinct profile shapes.

Person-centred investigations of digital competencies have also been reported in Korea. Kil et al. (2023) identified four latent digital literacy profiles among students, while Jeong and Choi (2024) identified three digital literacy profiles among high school students and reported differences in self-regulated learning, creativity, and academic achievement across profiles. These studies primarily examined general digital literacy and learning-related characteristics, whereas the present study focused specifically on generative AI literacy among adolescents with experience using generative AI. In addition, the present study extends prior profile research by examining academic self-efficacy and subjective well-being, alongside generative AI education experience. The observed differences in academic self-efficacy and subjective well-being remained significant after adjustment for gender, school level, academic achievement, and economic status, indicating that the profile differences persisted after adjustment for these measured background characteristics. These findings broaden the outcomes considered in person-centred research on AI-related literacy to include both academic and psychological dimensions, although the cross-sectional design precludes conclusions about the direction or causality of these associations.

Generative AI education experience also differed between the two literacy profiles. Adolescents in the Higher AI Literacy profile reported greater generative AI education experience than those in the Lower AI Literacy profile. UNESCO (2023) has emphasized the importance of education that enables students to understand the mechanisms, limitations, and ethical implications of generative AI, while Ng et al. (2021a, 2021b) have highlighted education as an important context for the development of AI literacy. Consistent with these perspectives, the present study found that greater generative AI education experience was associated with membership in the Higher AI Literacy profile. However, because the data were cross-sectional, the direction of this association cannot be determined. Greater educational exposure may be related to higher generative AI literacy, but adolescents with higher literacy may also be more likely to seek or engage with generative AI-related educational opportunities. Therefore, the present findings should not be interpreted as evidence that generative AI education causes higher literacy. Rather, they suggest that educational experience is an important correlate of generative AI literacy profiles and warrant further longitudinal or intervention research to determine whether structured educational experiences contribute to the development of information evaluation, communication, creative use, and ethical use competencies.

A substantial difference between the two profiles was observed in academic self-efficacy, and this difference remained significant after adjusting for gender, school level, academic achievement, and economic status. Self-efficacy refers to individuals’ beliefs in their capability to perform specific tasks (Bandura, 1997). In educational settings, academic self-efficacy reflects students’ confidence in successfully performing learning-related tasks and solving academic problems (Bulfone et al., 2020). One possible explanation for the present finding is that adolescents with higher generative AI literacy may be better able to use generative AI for information seeking, problem solving, and academic tasks, which may be associated with greater confidence when engaging in learning activities. This interpretation is consistent with Bećirović et al. (2025), who reported associations between AI literacy, self-efficacy, and academic performance, and with Hatlevik et al. (2018), who found a positive relationship between digital competence and ICT self-efficacy among students. The present findings therefore extend previous evidence by showing that differences in academic self-efficacy are also evident across generative AI literacy profiles and persist after adjustment for measured background characteristics. Nevertheless, the cross-sectional design does not establish whether higher generative AI literacy contributes to academic self-efficacy, whether adolescents with greater academic self-efficacy develop stronger generative AI literacy, or whether both are associated with other unmeasured factors.

Subjective well-being also differed significantly between the two generative AI literacy profiles, and the difference remained significant after adjusting for gender, school level, academic achievement, and economic status. Adolescents in the Higher AI Literacy profile reported higher subjective well-being than those in the Lower AI Literacy profile. This finding is consistent with Alshowkan and Shdaifat (2025), who reported a positive association between AI literacy and well-being, and is broadly compatible with longitudinal evidence linking adolescents’ happiness and academic achievement (Ham and Park, 2023). One possible interpretation is that adolescents who are more capable of evaluating, communicating with, creatively using, and ethically engaging with generative AI may experience greater confidence or perceived competence when navigating AI-supported learning environments. However, these mechanisms were not directly examined in the present study. The findings therefore indicate an association between generative AI literacy profiles and subjective well-being rather than evidence that higher generative AI literacy improves psychological well-being. Longitudinal and intervention studies are needed to clarify the direction of this association and the mechanisms that may account for it.

Taken together, the present study contributes to the emerging literature on generative AI literacy by applying a person-centred approach to examine variation across multiple literacy dimensions. Previous studies have often focused on average levels of AI literacy or associations among variables (Alshowkan and Shdaifat, 2025; Bećirović et al., 2025), whereas person-centred approaches provide a complementary means of examining how individuals may be grouped according to their patterns across multiple dimensions (Bergman and Trost, 2006; Howard and Hoffman, 2018). In the present study, however, the two identified profiles were differentiated primarily by their overall levels of generative AI literacy rather than by qualitatively distinct configurations across information evaluation, communication, creative use, and ethical use. Thus, the findings provide evidence of meaningful level-based variation in generative AI literacy but should not be interpreted as demonstrating sharply distinct types of adolescent AI users. Nevertheless, the differences in generative AI education experience, academic self-efficacy, and subjective well-being between the profiles suggest that variation in literacy level may be relevant when considering students’ experiences and support needs in AI-mediated educational environments. These findings may inform the development of educational approaches that consider differences in students’ generative AI literacy levels, while the effectiveness of differentiated support or instructional strategies should be evaluated in future longitudinal and intervention research.

Several limitations should be acknowledged. First, because this study employed a cross-sectional design, causal relationships among generative AI literacy, generative AI education experience, academic self-efficacy, and subjective well-being cannot be established. Longitudinal and intervention studies are needed to clarify the temporal and potentially causal relationships among these constructs. Second, all variables were measured using self-report questionnaires and may therefore be subject to response bias. In particular, generative AI literacy reflected adolescents’ self-perceived competencies rather than objectively assessed performance, which should be considered when interpreting the findings. Third, the analytical sample was restricted to adolescents who reported experience using generative AI; 1,819 adolescents without such experience were excluded. This restriction may have introduced selection bias because adolescents with generative AI experience may differ systematically from those without such experience. Consequently, the identified profiles and their associations with the study outcomes should not be generalized to adolescents without generative AI experience or to the broader adolescent population. In addition, because the data were collected from Korean adolescents, the findings may reflect characteristics of the Korean educational and sociocultural context, and their applicability to other populations and educational systems requires further examination. Fourth, although the original survey employed a stratified multistage cluster sampling design and provided sampling weights, the primary K-means clustering was conducted using unweighted data because the standard SPSS K-means procedure does not directly incorporate the survey design. Sensitivity analyses using the standardized sampling weight yielded substantively similar results; however, these analyses did not fully account for stratification and clustering in the sampling design. The population representativeness of the identified profiles should therefore be interpreted with caution. Fifth, the profiles were identified using K-means clustering, a distance-based approach that requires the number of clusters to be specified and may be sensitive to analytical decisions. Although alternative cluster solutions and cluster stability were evaluated, future studies could examine the reproducibility of the profiles using model-based approaches such as latent profile analysis and independent samples. Finally, the two profiles identified in this study differed primarily in their overall levels of generative AI literacy rather than displaying qualitatively distinct configurations across the four literacy dimensions. Accordingly, the profiles should be interpreted as level-based groupings rather than as discrete types of generative AI users, and future research should examine whether more differentiated profile structures emerge in other populations or with alternative measures.

## 5 Conclusion

This study identified two generative AI literacy profiles among adolescents with experience using generative AI: Higher AI Literacy and Lower AI Literacy. The profiles differed primarily in their overall levels of information evaluation, communication, creative use, and ethical use rather than in qualitatively distinct configurations across these dimensions. Adolescents in the Higher AI Literacy profile reported greater generative AI education experience and showed higher academic self-efficacy and subjective well-being, with the latter differences remaining significant after adjustment for background characteristics.

These findings highlight meaningful level-based variation in adolescents’ generative AI literacy and its associations with educational and psychological outcomes. They may inform educational approaches that consider differences in students’ generative AI literacy levels. Future longitudinal and intervention studies are needed to examine the stability of these profiles and clarify the temporal relationships between generative AI literacy and educational and psychological outcomes.

## Data Availability

Publicly available datasets were analyzed in this study. This data can be found here: https://www.nypi.re.kr/archive/mps.

## References

[ref1] Alshallal A. D. Alliott O. Brage S. van Sluijs E. M. F. Wilkinson P. Corder K. . (2024). Total and temporal patterning of physical activity in adolescents and associations with mental wellbeing. Int. J. Behav. Nutr. Phys. Act. 21:7. doi: 10.1186/s12966-023-01553-8, 38191365 PMC10775671

[ref2] Alshowkan A. Shdaifat E. (2025). Impact of AI literacy on well-being among nursing students: mediating roles of empowerment and anxiety: cross-sectional study. JMIR Nurs. 8:e79789. doi: 10.2196/79789, 41428941 PMC12721487

[ref3] Bandura A. (1997). Self-Efficacy: The Exercise of Control. New York: W. H. Freeman.

[ref4] Bećirović S. Polz E. Tinkel I. (2025). Exploring students’ AI literacy and its effects on their AI output quality, self-efficacy, and academic performance. Smart Learn. Environ. 12:29. doi: 10.1186/s40561-025-00384-3

[ref5] Bergman L. R. Trost K. (2006). The person-oriented versus the variable-oriented approach: are they complementary, opposites, or exploring different worlds? Merrill-Palmer Q. 52, 601–632. doi: 10.1353/mpq.2006.0023

[ref6] Bulfone G. Vellone E. Maurici M. Macale L. Alvaro R. (2020). Academic self-efficacy in bachelor-level nursing students: development and validation of a new instrument. J. Adv. Nurs. 76, 398–408. doi: 10.1111/jan.14226, 31588603

[ref7] Castelli L. Marcionetti J. (2024). Life satisfaction and school experience in adolescence: the impact of school supportiveness, peer belonging and the role of academic self-efficacy and victimization. Cogent Educ. 11:2338016. doi: 10.1080/2331186X.2024.2338016

[ref8] Cherewick M. Lama R. Rai R. P. Dukpa C. Mukhia D. Giri P. . (2024). Social support and self-efficacy during early adolescence: dual impact of protective and promotive links to mental health and wellbeing. PLOS Glob. Public Health 4:e0003904. doi: 10.1371/journal.pgph.0003904, 39739662 PMC11687749

[ref9] Ham Y. Park B.-H. (2023). A longitudinal analysis of the causal relationship between adolescents’ happiness, cooperation, and academic achievement: a multigroup analysis across genders. Stud. Korean Youth 34, 5–32. doi: 10.14816/sky.2023.34.2.5

[ref10] Hatlevik O. E. Throndsen I. Loi M. Gudmundsdottir G. B. (2018). Students’ ICT self-efficacy and computer and information literacy: determinants and relationships. Comput. Educ. 118, 107–119. doi: 10.1016/j.compedu.2017.11.011

[ref11] Howard M. C. Hoffman M. E. (2018). Variable-centered, person-centered, and person-specific approaches: where theory meets the method. Organ. Res. Methods 21, 846–876. doi: 10.1177/1094428117744021

[ref12] Jeong S.-J. Choi K.-U. (2024). Identifying digital literacy profiles of high school students: associations with self-regulated learning, creativity, and academic achievement. J/ Educ. Innovation Res 34, 325–348. doi: 10.21024/pnuedi.34.1.202403.325

[ref13] Kasneci E. Sessler K. Küchemann S. Bannert M. Dementieva D. Fischer F. . (2023). ChatGPT for good? On opportunities and challenges of large language models for education. Learn. Individ. Differ. 103:102274. doi: 10.1016/j.lindif.2023.102274

[ref14] Kil H. Park J. Kim H. (2023). Exploring students’ digital literacy and influencing factors using latent profile analysis: focusing on PISA 2018. Journal of Educational Culture 29, 351–374. doi: 10.24159/joec.2023.87.351

[ref15] Lee C. Mo S. Bae S. Lee S. (2024). Study on Adolescents' Use of Generative AI and Strategies for Enhancing AI Literacy (Research Report No. 24-Basic-02). Sejong: National Youth Policy Institute.

[ref16] Lee S. Park G. (2023). Exploring the impact of ChatGPT literacy on user satisfaction: the mediating role of user motivations. Cyberpsychol. Behav. Soc. Netw. 26, 913–918. doi: 10.1089/cyber.2023.0312, 38090765

[ref17] Long D. Magerko B. (2020). What is AI Literacy? Competencies and design Considerations Proceedings of the 2020 CHI Conference on Human Factors in Computing Systems. New York: Association for Computing Machinery, 1–16.

[ref18] MacQueen J. (1967) “Some methods for classification and analysis of multivariate observations,” Proceedings of the Fifth Berkeley Symposium on Mathematical Statistics and Probability Le Cam L. M. Neyman J. Volume 1, Berkeley: University of California Press, pages 281–297.

[ref19] Ng D. T. K. Leung J. K. L. Chu K. W. S. Qiao M. S. (2021a). AI literacy: definition, teaching, evaluation, and ethical issues. Proc. Assoc. Inf. Sci. Technol. 58, 504–509. doi: 10.1002/pra2.487

[ref20] Ng D. T. K. Leung J. K. L. Chu S. K. W. Qiao M. S. (2021b). Conceptualizing AI literacy: an exploratory review. Comput. Educ. Artif. Intell. 2:100041. doi: 10.1016/j.caeai.2021.100041

[ref21] Pajares F. (1996). Self-efficacy beliefs in academic settings. Rev. Educ. Res. 66, 543–578. doi: 10.3102/00346543066004543

[ref22] UNESCO (2023). Guidance for Generative AI in Education and Research. Paris: UNESCO.

[ref23] Wang D. Wang Q. Zhang S. (2026). Latent profiles of AI literacy among K–12 students: predictors and links to self-regulated learning. Front. Psychol. 17:1834851. doi: 10.3389/fpsyg.2026.1834851, 42325350 PMC13280791

